# Cross-cultural adaptation and content validation of the Nurses’ Professionalism Inventory to the Portuguese context: a methodological study

**DOI:** 10.3389/fpubh.2025.1473677

**Published:** 2025-02-14

**Authors:** Marlene Patrícia Ribeiro, Renata Cristina Gasparino, Olga Maria Pimenta Lopes Ribeiro

**Affiliations:** ^1^ICBAS, Abel Salazar Institute of Biomedical Sciences, University of Porto, Porto, Portugal; ^2^Tâmega and Sousa Local Health Unit, Penafiel, Portugal; ^3^School of Nursing, State University of Campinas, São Paulo, Brazil; ^4^Nursing School of Porto, Porto, Portugal; ^5^Center for Health Technology and Services Research (CINTESIS@RISE), Porto, Portugal

**Keywords:** nurses, professionalism, translating, validation study, professional practice

## Abstract

**Introduction:**

Nurses’ professionalism is linked to the profession’s principles and plays a crucial role in the quality and safety of care in the current context of complexity in healthcare systems. In the Portuguese context, no instrument allows evaluation of the nurses’ professionalism, which led to the objective of cross-culturally adapting and validating the content of the Nurses’ Professionalism Inventory for the Portuguese context.

**Methods:**

A methodological study was carried out and comprised six stages: translation, synthesis, back-translation, committee of experts, pre-test, and approval of the process by the original author developer of the instrument.

**Results:**

The first three stages were completed without difficulty. Nine experts were part of the committee to validate the instrument content. They evaluated items’ semantic, idiomatic, cultural, and conceptual equivalences, clarity, and relevance. Modified Kappa and Content Validity Index were calculated for all assessments. Seven items were submitted in two rounds to achieve consensus in the assessments. The usability of the instrument was tested in the fifth stage. Forty nurses performed the pre-test and agreed that the instructions, items, and response options of the Portuguese version of the Nurses’ Professionalism Inventory were easy to understand at 95.00, 97.50, and 92.50%, respectively. The author who developed the original instrument analyzed and approved the entire process carried out.

**Discussion:**

The cross-cultural adaptation of the Nurses’ Professionalism Inventory to the Portuguese context was carried out according to international recommendations, and the conclusion of this study will provide an instrument to assess the nurses’ professionalism in Portugal.

## 1 Introduction

Professionalism is a substantial component of all occupations, and it is defined as a set of beliefs about a profession’s roles and responsibilities (1, 2).

Professionalism in nursing is a fundamental multidimensional concept and a central aspect of nursing care (3, 4). It is manifested by the knowledge, attitudes, and behaviors that underlie competent and successful nursing practice (3, 5). Driven by socialization in formal nursing education, nurses’ professionalism is reflected in the interaction process, connoting the profession’s principles, care, and altruism (3).

Aspects related to the provision of care, such as ethical considerations, professional identity, professional growth, autonomy, and the complexity of relationships, are considered pillars of professionalism in nursing. They are closely interconnected and support the concept’s multidimensional structure (4).

Nurses’ levels of professionalism reflect commitment and identification with the profession (6), which can affect nurses’ autonomy, training, recognition, turnover, and the quality of care provided (3).

In the complex and rapidly evolving global healthcare landscape, nurses’ professionalism is underlined and plays a crucial role in ensuring the quality and safety of care (7) while upholding the values of integrity, responsibility, and respect (4).

The development of nurses’ professionalism is not their exclusive and individual responsibility. Nurse managers, such as organizations and society, must enhance it by creating positive nursing practice environments (8, 9). Therefore, evaluating the professionalism of nurses, in addition to being fundamental as a tool for nurse managers, is relevant since, resulting from this assessment, targeted interventions can boost the development of the profession and raise professional standards, resulting in a better quality of care, better results for patients, and a more positive image of nursing (10).

Currently, there are no validated instruments to assess nurses’ professionalism in Portugal. For this reason, in research and nursing management, instruments are used to evaluate nurses’ perceptions of their own skills (11) or their autonomy through other instruments (12). However, in addition to not allowing for the assessment of nurses’ professionalism, these evaluations may lead to considerations that pertain not only to nurses’ professionalism but also to other variables, such as the nursing work environment.

Furthermore, the development of the profession, both globally and in Portugal over the years, highlights aspects such as innovative vision, initiative for research, and evidence-based practice, which are valued from the perspective of a professional attitude that enhances the development of the nursing profession and discipline (13, 14).

Carefully crafted in the Japanese context, the Nurses’ Professionalism Inventory is a self-assessment measure of nurses’ professionalism (5). It underwent a thorough and critical review procedure, which ultimately developed five subscales: Accountability, Self-improvement, Professional attitude, Advancement of the nursing profession, and Professional membership (5). Furthermore, strong instrument performance was shown by the examination of the psychometric qualities (5, 10).

Given the importance of studying nurses’ professionalism and the lack of a validated instrument for evaluating it in Portugal, this study aims to perform a cross-cultural adaptation and content validation of the Nurses’ Professionalism Inventory (5) to the Portuguese context.

## 2 Materials and methods

**Figure 1:**
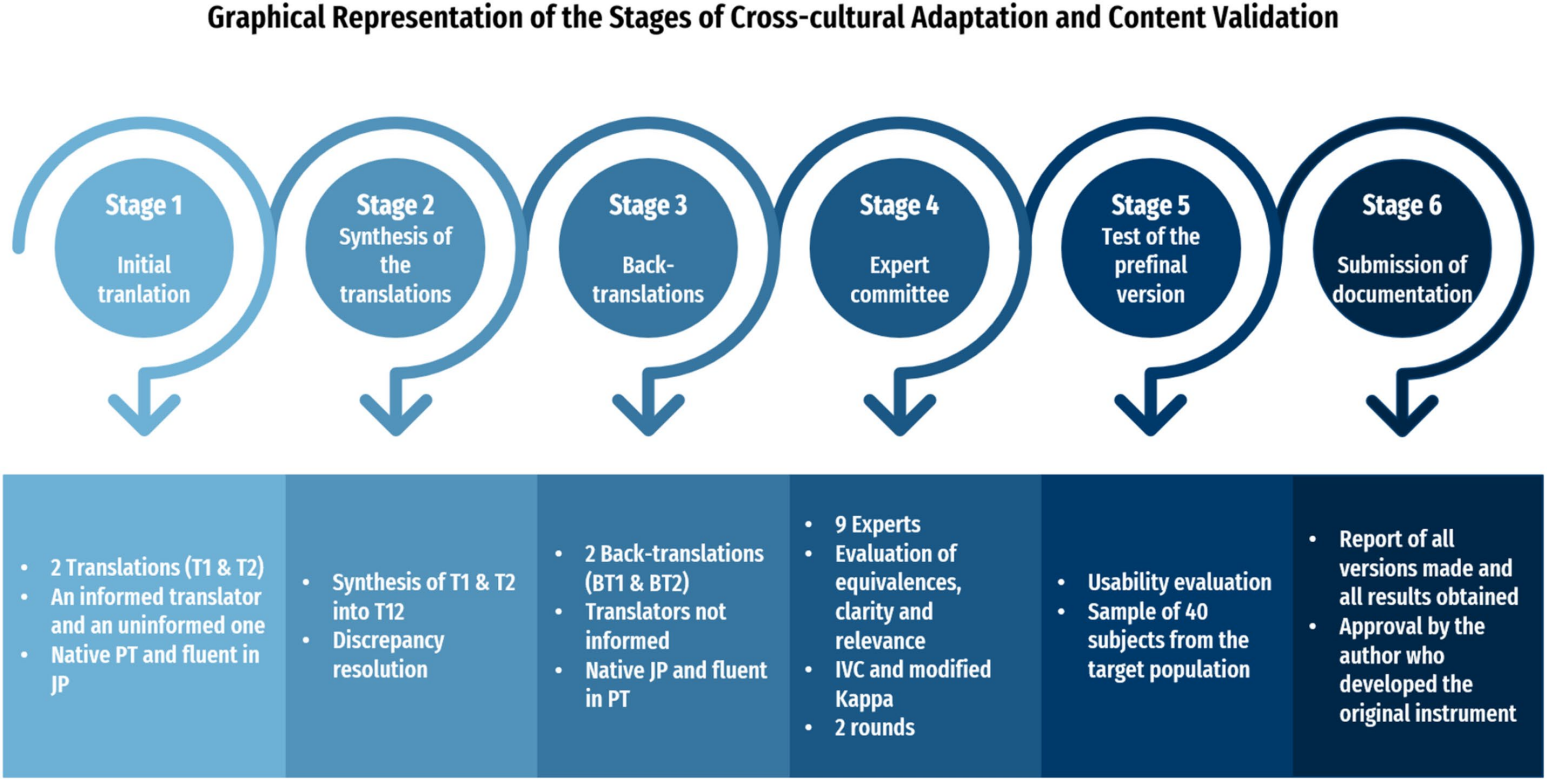
Graphical representation of the stages of cross-cultural adaptation and content validation.

This is a methodological study of the cross-cultural adaptation and content validation of the Nurses’ Professionalism Inventory (5) to the Portuguese context. It adheres to the internationally proposed recommendations by Beaton, Bombardier, Guillemin, and Ferraz for cross-cultural adaptation studies of measurement instruments, which include six stages: Initial translation, Synthesis of the translations, Back-translation, Expert committee, Test of the prefinal version, and Submission of documentation to the instrument developers (Figure 1) (15).

The cross-cultural adaptation of self-administered instruments intended for application in a different country, cultural context, and language demands a rigorous methodology. This approach encompasses not only a precise linguistic translation but also a thorough cultural adaptation, ensuring equivalence between the original and target versions, which are essential to safeguard the content validity and overall integrity of the instrument (15).

Research on the quality of such instruments highlights how their measurement properties are evaluated. Validity and reliability are regarded as the primary measurement properties. Consequently, after the cross-cultural adaptation and content validation phase for the Portuguese context, studies assessing these properties are essential in supporting the selection of valid and reliable instruments, thereby ensuring the robustness of study outcomes (16).

This methodological study of the cross-cultural adaptation and content validation to the Portuguese context was carried out between November 2023 and July 2024, and the process was started after permission from the author who developed the original version of the instrument.

### 2.1 Nurses’ Professionalism Inventory

The Nurses’ Professionalism Inventory development involved a review of self-improvement, the advancement of the nursing profession, professional attitudes, responsibility, and professional adherence. It also included interviewing seven registered nurses and holding short discussions with an external committee of eight participants, including physicians, journalists, and the head of a patient’s association (5, 10). The final version published consisted of a total of 28 items divided into five subscales: Accountability (8 items), Self-improvement (8 items), Professional attitude (6 items), Advancement of the nursing profession (4 items), and Professional membership (3 items) (5).

A Likert scale is employed for the questionnaire responses, graduated into six levels ranging from 1 to 6: “1 = Does not reflect anything” and “6 = Reflect completely.” This scale asks each participant to indicate to what extent the items reflect their awareness and behaviors concerning nursing (5).

The score for nurses’ professionalism corresponds to the sum of the values of the 28 items and can range between 28 and 168. Higher scores indicate more robust professionalism (5). The score for each subscale is calculated by summing the scores of the items that make up each subscale. So, subscales 1 and 2, Accountability and Self-improvement, are composed of eight items each; they can present scores between 8 and 48. Subscale 3, Professional attitude, contains five items. Therefore, scores can range between 5 and 30. Subscale 4, Advancement of nursing profession, comprises four items so that scores can be between 4 and 24. Subscale 5 can have scores between 3 and 18, comprising three items (5).

Instrument construction and validation revealed good psychometric properties in the Japanese context (5, 10). The confirmatory analysis demonstrated that the 5-factor structure is a good fit. Cronbach’s Alpha was used to analyze the internal consistency reliability, ranging from 0.84 to 0.90 (5).

### 2.2 Stage I: Initial translation

In the first stage, Initial translation, two forward translations (T1 & T2) were carried out independently from the original version of the Nurses’ Professionalism Inventory (Japanese) into the target language (Portuguese) by two native Portuguese translators residing in Portugal and fluent command of the Japanese language (5, 15, 17, 18).

The translator who carried out Translation 1 (T1) was contacted through the Japanese Embassy in Portugal, as this translator was identified in the teaching and training of the Japanese language in Portugal, with experience in Japanese-Portuguese and Portuguese-Japanese translation processes at the level of Japanese-Language Proficiency Test (JLPT) N1. This translator was informed about the translation’s concepts and objectives (15, 17, 18).

The translator who carried out Translation 2 (T2) was identified and contacted by a Multilingual Translation, Proofreading, and Certification Company. He has been translating and revising Japanese-Portuguese since 2011 and has worked as an interpreter for a Japanese company in Japan. Therefore, the proficiency level and the quality criteria required for the translation were ensured. This translator received no information about the translation’s concepts and objectives (15, 17, 18).

For both translations, difficulties or critical comments were reported within the process by each translator (15, 17, 18).

### 2.3 Stage II: Synthesis of the translations

In the second stage, Synthesis of the translations, the discrepancies and comments between T1 and T2 were analyzed and resolved between research team members, and a synthesis version of the two translations was carried out (T12) (15, 17, 18).

### 2.4 Stage III: Back-translation

The third stage, back-translation, was followed to provide quality and consistency to the translation. Two independent translators, natives and residents of Japan with fluent command of the Portuguese language, carried out two back-translations from the T12 synthesis version into Japanese. The two translators were identified and hired through the international connections of the Multilingual Translation, Proofreading, and Certification Company, ensuring the level of proficiency and the requirements necessary for the quality and certification of the translations. These two translators were blinded to the original version and unaware of the translation’s concepts and objectives. This stage led to two back-translations (BT1 & BT2), and both translators prepared a report with difficulties and comments confronted in the translation process (15, 17, 18).

### 2.5 Stage IV: Expert committee

In the fourth stage, an Expert committee evaluated the content validity of the synthesis version (T12) (15, 18). For convenience, nurses, nursing professors, methodologists, and translators with knowledge of the conceptual structure or the methodological process of translating and validating instruments constituted the committee of experts (15, 17, 18). A minimum of five experts with at least 4 years of experience and a master’s degree was considered (18, 19).

The form was presented online using the Google Forms® tool and consisted of two parts. Each item’s original, T1, T2, and T12 versions were presented in the first part. Experts were asked, after analyzing all versions, to evaluate the semantic (in the meaning of words), idiomatic (in colloquial expressions), cultural (in the cultural and experiential aspects of the target population), and conceptual (conceptual meaning under study) equivalences, as well as clarity (evaluating whether the item is perceptible, without the possibility of other interpretations) and relevance (assessing whether the item is relevant to evaluate the subscale concerned in the target context) (15–17). Regarding filling instructions and answer options, only the assessment of all equivalences and clarity was requested (15, 18).

The response options corresponded to a 4-point Likert scale. Therefore, for equivalences, the options were: (1) not equivalent, (2) needs major revision to be equivalent, (3) needs minor revision to be equivalent, and (4) equivalent. Regarding clarity, experts could choose: (1) not clear, (2) unclear, (3) clear, and (4) extremely clear. To assess the relevance, the options were: (1) irrelevant, (2) slightly relevant, (3) relevant, and (4) extremely relevant. For any answer marked as (1) or (2), experts were asked to present suggestions for improving the wording of the items (16, 18, 20).

The second part of the form consisted of closed-answer questions about the experts’ sociodemographic, professional, and academic characteristics.

The data obtained were organized using Microsoft Excel for Windows®. Simple descriptive statistical methods were applied to characterize the sociodemographic, professional, and academic characteristics.

Regarding content validity, data analysis at this stage comprised two approaches (17, 20). The first quantitative approach calculated the Content Validity Index (CVI) and Modified Kappa for all equivalences, clarity, and relevance (16–18). Considering the final number of experts on the committee (*n* = 9), it was established that the CVI should be equal to or greater than 0.90, and the Modified Kappa should be equal to or greater than 0.75 (16, 18, 20, 21).

Suggestions for improvement were integrated into a data file. Thus, a second qualitative analysis made it possible to improve the items whose evaluations did not obtain pre-established CVI and modified Kappa values (17). These items were enhanced, and a second round was conducted to reach an expert consensus. The changes led to a pre-final version being submitted to the fifth stage of the instrument’s cross-cultural adaptation process (15, 18).

At this stage, all experts consented to participate in a free, informed, and enlightened way (18).

### 2.6 Stage V: Test of the prefinal version

A pretest was conducted to evaluate the pre-final version’s usability, the average time needed to complete the questionnaire, and understanding of the instructions, items, and response options (15, 18). For this stage, a sample of between 30 and 40 subjects from the target population is recommended (15). Hence, a convenience sample of nurses and specialist nurses from the hospital context of a local health unit in northern Portugal, with a minimum of 1 year of experience, was recruited. Those who did not respond to the questionnaire in full were excluded. The questionnaire was administered online using the Google Forms® tool.

The questionnaire was composed of 3 parts. In the first, participants were asked to respond to the pre-final version of the instrument, recording the times they started and finished filling it out. In the second part, three questions to evaluate the instrument’s usability were asked regarding the ease of understanding the instructions for filling out the instrument, the items, and the response options. A 5-point Likert scale was employed for each of these questions: (1) strongly disagree, (2) disagree, (3) neither disagree nor agree, (4) agree, and (5) strongly agree. Participants who selected the options that reflected disagreement (1 or 2) in any of the three questions were requested to provide suggestions for improvement in an open-ended format. Finally, questions pertaining to the sample’s sociodemographic, professional, and academic characteristics were included in the third part.

The data obtained were organized using Microsoft Excel for Windows®, and simple descriptive statistical methods were applied to analyze them.

All participants in the fifth stage consented to participate in the study freely, in an informed, and enlightened way (18).

### 2.7 Stage VI: Submission of documentation to the instrument developers

In the sixth and final stage, the entire cross-cultural adaptation of the Nurses’ Professionalism Inventory (5) to the Portuguese context, which included all versions made and all results obtained, was reported to the developer of the original instrument (15, 18).

## 3 Results

Translators carried out the translation, synthesis, and back-translation stages without difficulty. Under the investigators’ supervision, all discrepancies were resolved.

Eleven potential participants were contacted to hold the expert committee, but only nine agreed to participate. The committee comprised nursing teachers, specialist nurses, a nurse manager, and one translator. All of them, except the translator, are involved in organizations, associations, or committees that promote the development of the nursing discipline and profession and present works developed and published in this context. Most have experience in the construction or cross-cultural adaptation of measuring instruments (55.56%). The translator reviewed all versions (T1, T2, T12, BT1, and BT2).

The minimum number of years of professional experience was 10 years. Other sociodemographic, professional, and academic characteristics are described in Table 1.

**Table 1:** Sociodemographic, professional, and academic characterization of the expert committee (*n* = 9).

Age (years) Mean; Std. Dev.	41; 6.88
Gender n (%)	
Female	5 (55.56)
Male	4 (44.44)
Marital status n (%)	
Married / non-marital partnership	7 (77.78)
Single	2 (22.22)
Education n (%)	
Master’s degree	8 (88.89)
Doctorate degree	1 (11.11)
Professional category n (%)	
Nursing teachers	4 (44.44)
Specialist nurse	3 (33.33)
Nurse manager	1 (11.11)
Translator	1 (11.11)
Time of professional practice (years) Mean; Std. Dev	18; 7.47

Source: Authors. Std. dev., Standard deviation.

In the first round, all items except items 6, 7, 11, 13, 18, 19, and 25 obtained CVI and modified kappa values greater than or equal to the pre-defined values of 0.90 and 0.75 for any of the assessments carried out.

The pre-established minimum CVI value was not reached in the evaluations of semantic equivalence and clarity of item 6; in the assessments of semantic, idiomatic, and conceptual equivalences, as well as the clarity of item 7; in assessing the relevance of item 11; in the assessments of all equivalences and clarity of item 13; in the evaluations of the idiomatic equivalence and relevance of item 18; in evaluating the clarity and relevance of item 19; and regarding item 25, in semantic and idiomatic equivalences and clarity (Table 2).

**Table 2:** Values of content validity index and modified Kappa for equivalences, clarity, and relevance evaluations for all instrument items.

Items	Semantic equivalence		Idiomatic equivalence		Cultural equivalence		Conceptual equivalence		Clarity		Relevance	
	CVI	Kappa	CVI	Kappa	CVI	Kappa	CVI	Kappa	CVI	Kappa	CVI	Kappa
1	1.00	1.00	1.00	1.00	1.00	1.00	1.00	1.00	1.00	1.00	1.00	1.00
2	1.00	1.00	1.00	1.00	1.00	1.00	1.00	1.00	1.00	1.00	1.00	1.00
3	1.00	1.00	1.00	1.00	1.00	1.00	1.00	1.00	1.00	1.00	1.00	1.00
4	1.00	1.00	1.00	1.00	1.00	1.00	1.00	1.00	1.00	1.00	1.00	1.00
5	1.00	1.00	1.00	1.00	1.00	1.00	1.00	1.00	1.00	1.00	1.00	1.00
6	**0.89**	0.89	1.00	1.00	1.00	1.00	1.00	1.00	**0.89**	0.89	1.00	1.00
7	**0.89**	0.89	**0.89**	0.89	1.00	1.00	**0.89**	0.89	**0.89**	0.89	1.00	1.00
8	1.00	1.00	1.00	1.00	1.00	1.00	1.00	1.00	1.00	1.00	1.00	1.00
9	1.00	1.00	1.00	1.00	1.00	1.00	1.00	1.00	1.00	1.00	1.00	1.00
10	1.00	1.00	1.00	1.00	1.00	1.00	1.00	1.00	1.00	1.00	1.00	1.00
11	1.00	1.00	1.00	1.00	1.00	1.00	1.00	1.00	1.00	1.00	**0.89**	0.89
12	1.00	1.00	1.00	1.00	1.00	1.00	1.00	1.00	1.00	1.00	1.00	1.00
13	**0.89**	0.89	**0.89**	0.89	**0.89**	0.89	**0.89**	0.89	**0.89**	0.89	1.00	1.00
14	1.00	1.00	1.00	1.00	1.00	1.00	1.00	1.00	1.00	1.00	1.00	1.00
15	1.00	1.00	1.00	1.00	1.00	1.00	1.00	1.00	1.00	1.00	1.00	1.00
16	1.00	1.00	1.00	1.00	1.00	1.00	1.00	1.00	1.00	1.00	1.00	1.00
17	1.00	1.00	1.00	1.00	1.00	1.00	1.00	1.00	1.00	1.00	1.00	1.00
18	1.00	1.00	**0.89**	0.89	1.00	1.00	1.00	1.00	1.00	1.00	**0.89**	0.89
19	1.00	1.00	1.00	1.00	1.00	1.00	1.00	1.00	**0.89**	0.89	**0.89**	0.89
20	1.00	1.00	1.00	1.00	1.00	1.00	1.00	1.00	1.00	1.00	1.00	1.00
21	1.00	1.00	1.00	1.00	1.00	1.00	1.00	1.00	1.00	1.00	1.00	1.00
22	1.00	1.00	1.00	1.00	1.00	1.00	1.00	1.00	1.00	1.00	1.00	1.00
23	1.00	1.00	1.00	1.00	1.00	1.00	1.00	1.00	1.00	1.00	1.00	1.00
24	1.00	1.00	1.00	1.00	1.00	1.00	1.00	1.00	1.00	1.00	1.00	1.00
25	**0.89**	0.89	**0.89**	0.89	1.00	1.00	1.00	1.00	**0.89**	0.89	1.00	1.00
26	1.00	1.00	1.00	1.00	1.00	1.00	1.00	1.00	1.00	1.00	1.00	1.00
27	1.00	1.00	1.00	1.00	1.00	1.00	1.00	1.00	1.00	1.00	1.00	1.00
28	1.00	1.00	1.00	1.00	1.00	1.00	1.00	1.00	1.00	1.00	1.00	1.00

Source: Authors. CVI, Content Validity Index; Kappa, modified Kappa. Bold values correspond to CVI values that did not reach the pre-defined value (0.90) in the first round.

Regarding these items, suggestions for improvement provided by experts were analyzed and led to changes in the wording of the items. Therefore, it was necessary to conduct a second round to evaluate all equivalences, clarity, and relevance of the new wording of items 6, 7, 11, 13, 18, 19, and 25. Consensus was achieved with the participation of all experts, as the new wording of items 6, 7, 11, 13, 18, 19, and 25 presented CVI and modified Kappa values of 1.00 for all assessments carried out (semantic, idiomatic, cultural and conceptual equivalences, clarity and relevance).

Experts suggested improving the grammar and writing of items 3, 5, 11, 14, 15, 20, 24, and 26. These suggestions were considered as they did not modify the content and improved the essay and the pre-final version of the instrument. The filling instructions presented CVI and modified Kappa values 1.00 for all equivalences and clarity.

At the end of the expert committee stage, a pre-final version of the instrument was obtained and subjected to a pre-test in the fifth stage. At this, the sample consisted of 40 participants, mainly female (67.50%), married or in a non-marital partnership (50.00%) and single (40.00%). The average age was 35 years (SD = 6.65; Min = 24; Max = 59). Regarding academic qualifications, 82.50% reported having a bachelor’s degree and 17.50% a master’s degree. The average time of professional nursing practice was 12 years (SD = 6.20; Min = 1; Max = 33), and 87.50% reported working as nurses and 12.50% as specialist nurses (Table 3).

**Table 3:** Sociodemographic, professional, and academic characterization of the sample participating in the pre-test stage (*n* = 40).

Age (years) Mean; Std. Dev.	35; 6.65
Gender n (%)	
Female	27 (67.50)
Male	13 (32.50)
Marital status n (%)	
Married / non-marital partnership	20 (50.00)
Single	16 (40.00)
Divorced	4 (10.00)
Education n (%)	
Bachelor’s degree	33 (82.50)
Master’s degree	7 (17.50)
Professional category n (%)	
Nurse	35 (87.50)
Specialist nurse	5 (12.50)
Time of professional nursing practice (years) Mean; Std. Dev	12; 6.20

Source: Authors. Std. dev., Standard deviation.

The average completion time was 5.08 min (SD = 1.97). Regarding understanding the instructions, items, and response options, participants agreed they were easy to understand at 95.00, 97.50, and 92.50%, respectively.

In the sixth stage, the Nurses’ Professionalism Inventory developer did not mention any particular difference between the content of the two back-translations (BT1 and BT2) and the original version. Furthermore, the developer approved the entire cross-cultural adaptation process and authorized its publication.

## 4 Discussion

This study focused on the transcultural adaptation and content validation of the Nurses’ Professionalism Inventory (5) for the Portuguese context and followed a rigorous methodological process (15) identified as one of the most used in studies of the transcultural adaptation of nursing and health instruments (18, 22). Methodological studies provide reliable and valid measurement instruments to the extent that cross-cultural equivalence is proportional to compliance with the methodological framework the authors propose using (22).

To reduce the difficulties inherent to the Japanese language, the first four stages of the process were accompanied by a translator knowledgeable about the process and concepts under study. Furthermore, the reports created by each translator involved in the translation and back-translation stages made it possible to ensure the content between the original and synthesis versions.

Although the back-translation stage (18) and the number of translators involved (17) are discussed, this study conducted the stage with two translators, according to the reference adopted. It should also be added that carrying out back-translations, in addition to allowing discrepancies in the translation to be identified, is helpful as a communication tool with the authors of the original version of the instrument (18).

The language and context of the origin of the Nurses’ Professionalism Inventory are not fluent and uncommon in the Portuguese context. Therefore, this is a way to check whether the meanings and contents between the original instrument and the translation into the target language convey the same meanings, thus ensuring the quality and consistency of the translation (17). For this reason, this critical step was carried out, and two back-translations were produced, matching the number of translations made, as seen in other similar studies (23–25).

Furthermore, the characteristics of translators were meticulously followed as recommended (15, 18). In the translation stage, translators were proficient in the language and culture of the source instrument, natives of the target language, independent and qualified, one informed and another uninformed. In the back-translation stage, there were two translators, native to the source language and fluent in the target language, independent and qualified, and both were not informed about the concepts and objectives of the translation (15, 18).

While this description is not consistent in some studies (26), other authors also precisely followed these recommendations to ensure the equivalence and content of the items between the original and adapted versions (23–25).

Regarding the fourth stage, for the constitution of the committee of experts, a minimum of five experts was considered, following what is suggested for transcultural adaptation studies of instruments, with at least a master’s degree of master and a minimum of 4 years of experience (15, 17–19). Furthermore, the committee’s composition included nursing professors, nurses, and a translator with knowledge of the methodological process or concept under study, as seen in similar studies (24, 27). However, the experts were selected intentionally since, in the Portuguese context, it is not easy to carry out sampling in any other way and comply with the established criteria. In other studies, the authors used search platforms and recruited a probabilistic sample, providing greater methodological rigor (24, 27).

As for how this stage was conducted, creating the online form made it possible to measure the CVI and modified Kappa for all equivalences, clarity, and relevance, as well as collect qualitative suggestions to improve the wording of the items. Another relevant aspect of this stage being carried out online was to ensure greater freedom for the experts’ opinions since they were not confronted with the physical presence of the researchers.

It should also be noted that the CVI value considered to obtain consensus was 0.90, which was recommended for a committee of more than six experts (18), which involved two rounds and allowed consensus to be obtained.

Despite the fact that some items presented a CVI greater than 0.90, the experts made suggestions for improvement that were implemented, such as harmonizing “practice” instead of “exercise” or, in item 26, changing “professional group” to “professional class,” improving idiomatic equivalence.

Another cultural aspect that raised doubts about the wording of the items was the references to “health professionals and social workers,” as the term health professional is used to encompass everyone colloquially in Portugal. However, according to the Portuguese Classification of Occupations (28), social workers do not belong to the Sub-Large Group “Health Professionals,” so the wording of the items according to T1, T2, and T12 was maintained.

This content validation method has generally been verified in other similar studies (23, 24, 27), but the authors do not provide transparent information or refer generically to its evaluation in some studies (25, 26).

In the pre-test stage, participants agreed that they understood the instructions, items, and response options, with values above 90%. No suggestions for improvement were received at this stage. The average time to complete the inventory was 5.08 min (SD = 1.97), which aligns with the recommendation that questionnaires should take less than 10 min for participants to complete and contain less than 50 items (29, 30). Furthermore, it suggests that the instrument is simple to apply and may involve participant adherence.

### 4.1 Implications for practice

The cross-cultural adaptation and content validation of the Nurses’ Professionalism Inventory to the Portuguese context will provide an instrument to assess the professionalism of nurses in Portugal, contributing to their global understanding in the respective context. Given nurses’ relevance in health systems, this knowledge will be fundamental to improving nursing training and promoting environments that support their professional development and growth.

Furthermore, health systems have been under pressure, and the COVID-19 pandemic has worsened this situation, so investing in nurses’ professionalism is essential for resilient and effective public health responses.

### 4.2 Limitations

Despite the relevance of the study, it has some limitations. As a limitation of the present study, the impossibility of all experts on the committee to interpret the back-translations is mentioned since most of them did not command the Japanese language, which is not common in the Portuguese context.

Moreover, the study focuses on the first phase of a cross-cultural adaptation and content validation of the Nurses’ Professionalism Inventory (5) to the Portuguese context, recognizing as a limitation that other measurement properties were not evaluated. Therefore, as a suggestion for future research, it is recommended that other psychometric properties, such as internal consistency or reliability, be investigated.

## 5 Conclusion

The cross-cultural adaptation and content validation of the Nurses’ Professionalism Inventory to the Portuguese context followed international recommendations most used in similar studies. The results demonstrated evidence of the content validity and usability of the Portuguese version of the instrument.

Although the Japanese cultural context is not very close to the Portuguese cultural context, the development of the nursing profession and what is expected of nurses as professionals are similar, meaning that the item’s content of the Nurses’ Professionalism Inventory reflects the professionalism of nurses in both contexts.

## Data Availability

The raw data supporting the conclusions of this article will be made available by the authors, without undue reservation.
